# Family Planning Practices in Appalachia: Focus Group Perspectives on Service Needs in the Context of Regional Substance Abuse

**DOI:** 10.3390/ijerph17041198

**Published:** 2020-02-13

**Authors:** Laura E. T. Swan, Samantha L. Auerbach, Gretchen E. Ely, Kafuli Agbemenu, Jessica Mencia, Nimah R. Araf

**Affiliations:** 1 School of Social Work, Virginia Commonwealth University, Richmond, Virginia, VA 23284, USA; 2 School of Nursing, University at Buffalo, Buffalo, New York, NY 14260, USA; slauerba@buffalo.edu (S.L.A.); agbemenu@buffalo.edu (K.A.); 3 School of Social Work, University at Buffalo, Buffalo, New York, NY 14260, USA; geely@buffalo.edu (G.E.E.); jjmencia@buffalo.edu (J.M.); 4 Department of Psychology, University at Buffalo, Buffalo, New York, NY 14260, USA; nimahara@buffalo.edu

**Keywords:** reproductive health, pregnancy, contraception, rural health, opioid use, healthcare access, Appalachia

## Abstract

Reproductive health disparities in the Appalachian region may be driven by barriers to healthcare access. However, the barriers specific to accessing family planning services in Appalachia have not yet been identified from the perspectives of Appalachian community members. Moreover, it is unclear how community members might perceive elevated levels of opioid use in the region to impact family planning practices. To fill this gap in knowledge, the current qualitative study explored community perspectives about family planning in Appalachia in the context of the opioid epidemic for the purpose of developing a survey instrument based on these responses. We conducted three video call focus group interviews with community stakeholders, those who live, work and are invested in Appalachia (*N* = 16), and analyzed the responses using Levesque, Harris, and Russell’s (2013) five pillars of healthcare access as a framework to categorize family planning practices and perceptions of service needs in the context of regional substance abuse: (1) approachability, (2) acceptability, (3) availability and accommodation, (4) affordability, and (5) appropriateness. Subthemes within each of these five categories were also identified. Our findings highlight stakeholder concerns around a lack of knowledge about and access to family planning services in Appalachia. Community members also expressed concern around the lack of availability of substance use treatment services, which may negatively impact family planning use and access in the region.

## 1. Introduction

Ensuring access to reproductive healthcare, which includes family planning services, is essential to meeting public health goals set forth by the World Health Organization, United Nations, and Centers for Disease Control and Prevention [1,2,3]. Healthcare access is multi-dimensional, however, and can be impacted by individual-level factors such as the perceived need for care and community-level factors such as the availability of healthcare providers [4]. While we are not aware of a resource that details unintended pregnancy rates, family planning practices, or the availability of reproductive health services in the overall Appalachian region, research does indicate that there are barriers to general healthcare access in the region, which include problems accessing providers and facilities, problems covering healthcare costs, and a lack of health insurance coverage [5]. Moreover, experts indicate that substance use is known to negatively affect women during their reproductive years, which suggests that family planning practices may be impacted by individual substance use and levels of substance use in the community [6].

Given that Appalachian counties are located across many states, problems with healthcare access vary throughout the region, as public health policy, including access to public health insurance plans, varies by state. For example, Tennessee did not expand Medicaid access under the Affordable Care Act, while New York State did [7]. As a result, access to health insurance coverage in the Appalachian counties in these states will differ, thus impacting the affordability of healthcare, even when services are available.

### 1.1. Background

#### 1.1.1. The Appalachian Region

Appalachia is a large geographic region in the Eastern United States, following the path of the Appalachian Mountain range and encompassing the entire state of West Virginia, almost all of Pennsylvania, and parts of Alabama, Georgia, Kentucky, Maryland, Mississippi, New York, North Carolina, Ohio, South Carolina, Tennessee, and Virginia [8]. This region is home to more than 25 million people in 420 counties across these 13 states [8]. The region is comprised of five subregions: (1) Northern Appalachia, which includes the Appalachian counties in New York, Pennsylvania, Maryland, and some of Ohio; (2) North Central Appalachia, which includes the rest of the Ohio Appalachian counties, and most of the counties in West Virginia; (3) Central Appalachia, which includes all the Appalachian counties in Kentucky, some in Tennessee, and a handful in Virginia and West Virginia; (4) South Central Appalachia, which includes all the Appalachian counties in North Carolina, the majority of the Tennessee Appalachian counties, and some in Virginia and West Virginia; and (5) Southern Appalachia, which includes all the Appalachian counties in Alabama, Georgia, Mississippi, and South Carolina [9]. These subregions represent contiguous areas that share common topography, demographics, and economics [9].

The Appalachian region contains rural areas, urban clusters, and urbanized areas, which are categorized based on population density [10]. Currently, the Census Bureau classifies *urbanized areas* as any incorporated area with a population of 50,000 or more, classifies an *urban cluster* as an incorporated area with a population of at least 2500 and less than 50,000, and classifies a *rural* area as any area that falls outside of the urban classifications [10]. Based on these classifications, 42% of the Appalachian region’s population lives in areas classified as rural; this is more than double the percentage of people living in rural areas across the United States as a whole [8].

#### 1.1.2. Appalachian Culture

Despite the existence of both urban and rural areas within the large Appalachian geographic area, the proximity of the region to the Appalachian mountain range unifies the region. The notion of an Appalachian cultural identity dates back to the 1800s, and it was originally centered around an attempt to better understand the people of the region who were known to be distinct from surrounding populations [11]. Thus, the Appalachian Regional Commission characterizes the region collectively, in order to highlight the shared uniqueness and common problems of the region, based on an understanding that Appalachian residents share some experiences whether or not they reside in urban or rural areas [8]. Despite the existence of subregions, which may have unique cultures, it is understood by those familiar with the region (and for the purposes of our research, we have adopted this understanding), that the region shares common elements of an overall culture.

Among other commonalities, this culture is linked around navigating stereotypes, proximity to the mountains and geographic isolation, economic distress and poverty, shared cuisine and music, and exploitation of natural resources [8,10,11]. To further support the existence of an overall Appalachian culture, it has been argued that the Appalachian population is a vulnerable population comparable to other distressed, under-represented minority populations [12]. Experts also note that subcultures exist, which would be expected in such a large geographic area [13]. Negative stereotypes (e.g., Appalachians as an under-educated population) often predominate perceptions of Appalachian culture, while ignoring “resilience, pride and self-sufficiency” [13] (p. 95) and a strong values system [14] as regional strengths. In light of research suggesting that cultural factors influence health and that culture plays a role in both health behaviors and health outcomes in Appalachia [15], the purpose of our research was to learn more about family planning practices from the perceptions of community members who are immersed in this culture.

#### 1.1.3. Health Disparities

Existing research suggests that Appalachian residents face a multitude of health disparities compared to Americans living in other regions. For example, rates of cancer incidence and mortality [16], heart disease [17], obesity [18], diabetes [19], smoking [20], and drug use [21] are all higher in Appalachia compared to other US regions, and those living in Appalachia are at greater risk of low health literacy [22] and perceive their overall health status as poorer than those living in other areas [23].

People living in Appalachia often face barriers to accessing health services including long wait times at healthcare provider offices [24], difficulty accessing clinics during operating hours [12,14], and difficulty obtaining transportation to health services [12,15]. The cost of health services can be prohibitive, especially for community members living in poverty and those lacking health insurance [15,16,17,18].

Appalachians’ shared cultural norms may impact their ability to access care. Fatalism about health, for example, as evidenced by perceptions that health outcomes are out of one’s personal control, and/or are in “God’s hands,” is a cultural perception that research suggests is common in the region [25] (p. 109). Appalachian community members sometimes lack trust in their healthcare providers [16,19,20,21,22], perceive cultural differences between themselves and their providers [14,22,23], and/or feel intimidated or embarrassed about seeking care [15,24], which can encourage them to seek health advice from other sources, such as from the internet [26] and from religious leaders [27].

Evidence indicates that Appalachian counties have higher healthcare costs when compared to other counties within their states [5]. Insurance coverage, however, is also higher in these counties, which is attributed to the high rates of participation in Medicare disability and Medicaid in the region [5]. As noted above, access to the programs varies by state, as a result of variants in state public health policy, including whether or not states chose to expand Medicaid under the Affordable Care Act [5].

#### 1.1.4. Appalachian Family Planning Amid the Opioid Epidemic

##### Reproductive Health and Family Planning Disparities

Among the health disparities faced by Appalachian residents are disparities in access to family planning and reproductive healthcare. Research from the early 1990s indicates that the fertility rate in Appalachia was falling at that time, suggesting that the population was in support of fertility regulation, which potentially translates into the assumption that contemporary Appalachians may be generally open to contraceptive use [28]. Recent research indicates that rates of unintended pregnancy in an Appalachian sample are elevated, which has been attributed to the use of predominately user-dependent contraceptives with high failure rates [29]. In the same study, women were more likely to be interested in obtaining long-acting reversible contraception (LARCs) if they had insurance coverage that allowed them to pay $200 or less for an intrauterine device (IUD), which is a method of contraception that is not user-dependent and is thus highly effective [29]. Experts note that Appalachian cultural values that include traditional gender norms contribute to stigmatized perceptions of sexuality in the region [30], which may impact contraceptive choices and other family planning practices.

In 2017, Appalachian births accounted for approximately 7% of all US births [31]. Additionally, maternal characteristics have been shown to vary between the Appalachian region and the rest of the United States [31]. For example, in comparison to the rest of the United States, infants born in Appalachia are more likely to have mothers who are younger and less educated, and they are more likely to die in the first year of life [31]. Another study found that babies born in rural Central Appalachian counties were more likely to be preterm and low birth weight, on average, compared to those born in more urban counties, and this finding holds true in the greater Appalachian region, as well [29,30].

Of particular concern is the elevated rate of neonatal abstinence syndrome (NAS), a post-birth infant drug withdrawal syndrome, in the Appalachian region [32]. NAS has disproportionately affected the Appalachian region, which shows the highest incidence of NAS in the country, with 33.4 cases per 1000 births [32]. Findings from one study indicated that NAS cases in rural and Appalachian Kentucky counties were approximately two and a half times higher than rates in urban and non-Appalachian Kentucky counties [33]. In another study, researchers referred to NAS as an epidemic in the Eastern Tennessee region of Appalachia, where NAS cases accounted for 28.8 out of 1000 live births in 2014 [34].

##### Opioid Use

While substance abuse across the United States is a common social problem, rates of opioid use in the US have risen sharply in the last decade, and Appalachia has been hit hard by the opioid epidemic. For example, West Virginia, which is the only state located entirely in the Appalachian region, has an opioid drug overdose death rate of 49.6 deaths per 100,000 people, which is threefold higher than the national rate of 14.6 [32]. While we are unable to determine the extent of resources available for substance use treatment in the Appalachian region overall, experts do note that the substance use treatment infrastructure in rural Appalachia is limited [35].

##### Family Planning and Opioid Use

Elevated rates of opioid use in the region likely intersect with family planning practices in a variety of ways. In light of research indicating that pregnant women avoid seeking health services to minimize the risk of exposing their drug use [36], it is likely that Appalachian individuals who are using opioids may avoid seeking family planning services out of fear that their drug use will be revealed to health or criminal justice professionals. Therefore, the opioid epidemic may further complicate the use of family planning services, which are already stigmatized in the region [30]. However, one obvious benefit of family planning during active drug use is the reduction of NAS, which, as noted above, is at epidemic levels in the Appalachian region.

While there is statistical information available about certain health conditions across Appalachia, including birth outcome data [31], to our knowledge there is no survey of family planning practices for the region. Moreover, extant research does not examine family planning from the perspectives of community stakeholders. Finally, information about how the opioid epidemic is impacting family planning from a community stakeholder perspective is lacking. Thus, the purpose of this study was to conduct focus groups to inform future survey development through the exploration of community stakeholder perceptions about family planning practices in the Appalachian region and to discover how community members perceive the opioid epidemic to intersect with family planning in the region.

#### 1.1.5. Healthcare Access Framework

As Appalachia is a region with historically-impeded access to healthcare [37], we utilized Levesque, Harris, and Russell’s (2013) healthcare access framework to structure our analysis and results [4]. This framework can be used to conceptualize access to healthcare, arguing that there are five dimensions of healthcare accessibility: (1) approachability, (2) acceptability, (3) availability and accommodation, (4) affordability, and (5) appropriateness. Together, these dimensions generate a holistic view of healthcare access. Approachability refers to a person’s ability to perceive the need for healthcare; acceptability relates to a person’s ability to seek healthcare; availability and accommodation influence a person’s ability to reach healthcare; affordability refers to a person’s ability to pay for healthcare; and appropriateness influences a person’s ability to actively engage in healthcare. Each of these dimensions of healthcare access also represents an area where access can be obstructed, allowing a potential gap in health services to emerge. This study applied this framework of healthcare access to family planning in Appalachia, in the context of the opioid epidemic, allowing for the exploration of the healthcare needs of this population.

## 2. Materials and Methods

### 2.1. Sample

Participants were recruited from professional networks using purposive sampling and were compensated for their time with $50 retail gift cards. All participants (*N* = 16) across the three focus groups were Appalachian residents and community stakeholders employed in professional settings, including healthcare, social services, and university settings. These were people who live and work in Appalachian zip codes and who self-identify as Appalachian people with a stake in the health of the community and in the well-being of Appalachia as a whole. Participants were from Appalachian regions of Kentucky (*n* = 6), Tennessee (*n* = 4), West Virginia (*n* = 2), North Carolina (*n* = 2), South Carolina (*n* = 1), and Pennsylvania (*n* = 1). One participant identified as transgender, and the remaining 15 participants identified as cisgender women. Ages and ethnic backgrounds were not requested from participants, as the only requirement for participation was current stakeholder status in Appalachia. Participants did not have to hold any expertise in family planning, as we were seeking community input, not necessarily experts on the topic. While these groups did not contain participants from every Appalachian state, all of the Appalachian subregions were represented, and the comments from the participants were similar across focus groups, suggesting adequate information power [38].

### 2.2. Data Collection

We conducted the three focus group interviews remotely, via the Zoom video call platform. The interviews were guided with structured questions designed to elicit information about respondents’ perspectives with the purpose of generating community-informed questions for the development of a culturally-appropriate, quantitative measurement tool for the future study of regional family planning practices and the impact of community opioid use on family planning in the region. Participants were told that their answers would be used both to learn about the needs of their community and to inform the development of the survey. Participants were asked guiding questions about their perceptions around important issues in family planning, community substance use, community reproductive health services, and how the elevated levels of opioid use in their communities might be impacting family planning practices in the Appalachian region. The focus groups were recorded and then professionally transcribed. This research protocol was reviewed and approved by the Institutional Review Board at the institution where the study originated.

### 2.3. Data Analysis

To analyze the data, we used the principles of Braun and Clark’s (2006; 2012) approach to thematic analysis which involves a six-step process: (1) becoming familiar with the data, (2) generating initial codes, (3) searching for themes, (4) reviewing themes, (5) defining and naming themes, and (6) producing the report [39,40]. As we became familiar with the data, we coded the comments that represented the five a priori codes, which were taken from Levesque, Harris, and Russell’s (2013) five dimensions of healthcare accessibility [4]. As expected, we found that all responses fit into one of these five codes. Additionally, subthemes emerged during coding, and we defined and named these subthemes, categorizing them in relation to the general a priori themes.

A team of two researchers double-coded the first five pages of the first focus group, coding all participant responses into one of the five initial themes, and then met with a third member of the research team to compare codes and discuss the coding process, in order to ensure inter-rater reliability. We then created a codebook, which was adapted from Levesque et al. (2013) [4] and double-coded all three focus group interview transcripts to ensure accurate coding and agreement among the research team, meeting with a third research team member after completing coding of each of the three transcripts to reconcile any discrepancies around the codes. Any discrepancies in the coding selections served as discussion points among the three-person research team. After coding all three transcripts, we worked as a research team to identify and label subthemes within each of the five a priori categories.

## 3. Results

**Figure 1:**
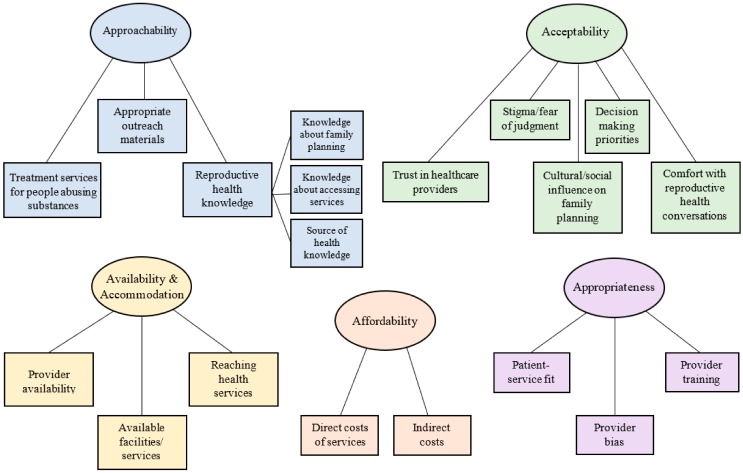
Thematic Map for Family Planning Needs in Appalachia.

Our findings, which are discussed below, are categorized into the five overarching themes that represent the aspects of healthcare access conceptualized by Levesque, Harris, and Russell (2013) [4], as well as subthemes that emerged within each of these overarching categories. A graphic representation of these findings is shown in a thematic map in Figure 1, and a description of the a priori themes alongside the subthemes we identified are shown in Table 1.

The five overarching themes (approachability/ability to perceive the need for care; acceptability/ability to seek care; availability and accommodation/ability to reach care; affordability/ability to pay for care; and appropriateness/ability to engage in care), which are described in detail below, are distinct from one another, although they also contain overlapping elements. For example, while the availability/accommodation and affordability themes both refer to transportation as a potential barrier to care, the affordability theme refers specifically to the ability to afford the costs of care, including expenses related to transportation to care while the availability/accommodation theme refers specifically to being able to be physically present at a health facility, where convenience and availability of services are largely the barriers to care. 

### 3.1. Approachability

The approachability theme referred to community members’ ability to perceive the need for health services and to identify that such services exist and can impact people’s health. Quotes coded as approachability mentioned transparency of information, provider outreach activities, screening procedures, or patient health literacy or expectations as issues that stakeholders believed would prevent Appalachian people in their own communities from being able to perceive the need for healthcare and therefore be able to approach family planning services. These issues related to community members being able to identify that a form of service exists and is important for their health.

#### 3.1.1. Treatment Services for People Abusing Substances

The treatment services for people abusing substances subtheme captured stakeholder comments about Appalachian people who abuse substances, particularly opioids, being able to perceive the need for family planning and substance abuse treatment services. One participant mentioned that community members who need substance abuse treatment may not know what services to pursue if they were to get pregnant, stating that it is necessary to ask patients if they “have a plan for addiction recovery if [they] do become pregnant, because there’s different options…Is there a plan with it, and do they seek [treatment] options throughout the plan?”

Another participant commented that sometimes treatment services for people using substances focus almost solely on drug treatment, while other services, including contraception, are an afterthought, which can lead to unplanned pregnancies, especially in circumstances when community members may be trading sex for drugs or money. This participant stated:

*Like that pattern of the drug-seeking being the primary focus and birth control is not an issue. And sometimes that means I get high, and I had sex, and end up pregnant or sometimes I had sex to get my drugs, then I end up pregnant.*

Participants also discussed the impact of NAS in the Appalachian region, commenting on the importance of spreading information to help educate the public and prevent NAS cases. One participant stated, “there’s a huge awareness piece right now in the [blank] County area about reducing neonatal abstinence syndrome, the impact of neonatal abstinence syndrome.” Similarly, another stakeholder commented, “I think this region [in Appalachia], in particular, is really becoming very aware about neonatal abstinence syndrome in correlation to substance use and family planning. A lot of people are trying to help with NAS in a variety of different ways.” These quotes demonstrate that stakeholders in Appalachia have identified a lack of information as an issue related to high NAS rates and are working to provide information to this community, which would allow community members to perceive the need for certain family planning and/or substance use services.

#### 3.1.2. Appropriate Outreach Materials

Stakeholders also discussed the importance of healthcare providers disseminating appropriate reproductive health outreach materials to the community, in order to help them perceive the need for contraception and family planning care. Specifically, participants commented on the importance of outreach materials matching the reading and health literacy level of the community members they are trying to reach. One participant described working with a health literacy team to ensure that outreach materials were appropriate, stating:

*I know for a lot of patient education materials I’ve had to create, we have connected with our health literacy team, and we realized that we were writing education materials at a college reading level, and they helped us change wording around to get it closer to that target 6th-grade reading level.*

Another participant reiterated this comment, describing the importance of ensuring that “the material provided to [community members] for educational purposes [is] understandable to them.”

Participants also connected this issue to community members in need of both substance use and family planning services, describing the need to assess whether a “written handout for education, was…understandable as it relates to [both] addiction and carrying a pregnancy. The risks, benefits of going to something like Subutex. [Do] they feel like they got good information to make decisions, and understand?”

#### 3.1.3. Reproductive Health Knowledge

Many participants described how a lack of reproductive health knowledge can be a barrier to Appalachian community members’ ability to perceive the need for health services. The stakeholders explained that knowledge about family planning, knowledge about accessing health services, and the source of health information are all important aspects of reproductive health knowledge. As shown in the quotes below, the stakeholders described how, in order to perceive the need for healthcare and the effectiveness of health services, Appalachian community members need to understand the concept of family planning and the purpose of reproductive health services and be able to identify how to access such services. Additionally, the source and frequency of exposure to this information are important, because different sources have different perceived authority and motives, which may impact a community member’s ability to accurately perceive the need for services.

##### Knowledge about Family Planning

Participants explained that, in order to understand Appalachian community members’ ability to perceive the need for health services, it is important to begin by understanding what the term “family planning” means to these community members. For example, several participants commented that the meaning of the term “family planning” may not be clear to all Appalachian community members, with one participant stating:

*I don’t think a lot of people even know what family planning is or the definition of that, cause that’s not language they hear or [were] taught in school or at home. So you know, what we consider family planning, I don’t think would ever come across their mind as family planning.*

Other participants extended this comment to community members who may fail to realize that family planning means more than birth control, with one participant stating:

*When students do speak about it to me, or even just people in general in the community, it’s usually just birth control. That’s all I hear about, the birth control aspect. And it’s not saying not wanting to get pregnant, it’s just always just birth control. They don’t talk about safe sex, they don’t talk about STD testing or all these different things that they could receive at a family planning clinic or at a Planned Parenthood; they just think birth control or abortion, automatically.*

Participants also shared stories about their own experiences and those of other Appalachian people. One stakeholder shared that they had worked with 18- and 19-year old college women who:

*…didn’t know basic health, reproductive health…they don’t even know….that birth control can be used for more than just preventing pregnancy…I had several girls that did not realize that birth control can be used to help with medical conditions related to reproductive health.*

These quotes emphasize the need for community members to learn about reproductive health and family planning and understand that it encompasses multiple options in order to perceive the need for these health services.

##### Knowledge about Accessing Services

The stakeholders also identified knowledge about accessing health services as an important factor in community members’ ability to perceive the need for healthcare. One participant commented that it is important to better understand whether people “know where to go to…access family planning [services].” Another participant built on this comment as it pertains to accessing abortion care, questioning whether Appalachian community members:

*…even know where to go [for abortions]? Do they even know there [are] two forms? There’s the surgical, and then there’s the abortion pill. Cause now, people really confuse…Plan B with the abortion pill. They tend to go to Walmart and [try] get [the abortion pill] over the counter, and it’s like no. That’s not the way it works.*

##### Source of Health Information

The stakeholders also indicated that the source of health information plays an important role in Appalachian community members being able to perceive the need for healthcare. Participants emphasized the different sources of reproductive health information, specifically mentioning that Appalachian community members might learn about reproductive health from friends, family, church, religion, parents, the internet, the health department, school-based sexual education, or crisis pregnancy centers. One participant stated that it is important to determine where people “…learn about birth control. I find as a clinician that when I run through the range of options, often women have preconceived ideas of what’s okay or not based on friends’ experience.” Similarly, another participant discussed school-based sexual education, focusing on the number of times that people are exposed to reproductive health information. This participant stated the importance of:

*…how many times are they getting [information], cause the thing that comes back to kind of the source is how many sources are you getting it from? If you’re only getting it one time from one source then we need to think about are we really giving them a broad...more options. Cause typically, it’s usually they hear it one time, and that’s it.*

### 3.2. Acceptability

The acceptability theme referred to community members’ ability to seek or pursue healthcare. Quotes categorized under this theme mentioned personal or community values, social norms, culture, or gender as issues that kept people from seeking healthcare services. These issues related to cultural and social factors that determined Appalachian community members’ ability to accept aspects of health services and service providers, such that they choose to pursue healthcare.

#### 3.2.1. Trust in Healthcare Providers

The trust in healthcare providers subtheme captured information about how trust or lack of trust in healthcare providers may influence a community members’ ability to seek care. This issue is related to provider outreach regarding the types of services they provide, community members’ comfortability discussing health issues with a provider, and the confidentiality of services. The stakeholders reported that without these pieces present, community members may lack trust in their providers, which can influence peoples’ ability to seek family planning services. One participant reported that community members have trouble opening up to healthcare providers whom they do not trust because they fear getting a lecture. That participant stated:

*I think people don’t feel like they can trust their provider…they’re always gonna get that lecture. They’re always gonna get that talk, you know. ‘Why [are] you sexually active?,’ ‘Why [are] you doing this?,’ Or, ‘why [are] you doing that?’ So just that level of trust.*

Another participant commented on issues of trust that arise in small, rural communities, where community members may avoid acknowledging their health issues out of fear that their healthcare provider will not keep their treatment confidential. This stakeholder commented that research with this population should ask community members:

*…‘do you have any providers that you trust in your community?’ And, ‘do you trust the confidentiality of those services?’ Because there are a lot of small communities around here, and [people might wonder,] ‘if I go to the health department is everybody going to know I have hep C?’*

Participants also discussed trust in healthcare providers as it pertained to the intersection of family planning and substance use issues, noting that some Appalachian community members fear that providers may betray their trust by reporting their substance use during pregnancy to authorities. One participant stated:

*…there’s a lot of fear around talking about pregnancy and drug use together. What that could mean for themselves, and if they’re going to be in danger. And I think it’s pretty similar to other areas of, you hear anything about pregnancy or reproductive health and drug use together, and you get nervous. I think there’s a lot of fear around this.*

Community stakeholders noted that such a lack of trust in healthcare providers can keep members of their community who use substances from understanding and seeking care related to pregnancy.

#### 3.2.2. Stigma/Fear of Judgment

The stigma/fear of judgment subtheme captured participants’ comments related to barriers to Appalachian community members seeking care because of the stigmatized nature of their health concerns, which can lead to a fear of being judged by healthcare providers or others in the community. Participants discussed stigma in Appalachia related to sex and reproductive health topics, with one participant stating:

*I think a problem with this area is people choose not to go [to the doctor] because they feel like they’re really judged because they’re going and asking those [reproductive health] questions, then they know they’re gonna get a very judgmental body language back. Or just an answer back that is not an answer they need.*

Participants felt that fear of discussing stigmatized health topics with a healthcare provider prevented Appalachian community members from seeking care.

The stakeholders also discussed the stigmatized nature of opioid use, especially among pregnant community members. For these people, seeking care may be especially frightening because they fear both provider judgment and possible legal action. One participant stated:

*One of the biggest issues, especially, with the opioid recovery, is…them not coming in for initial family planning, but also when they do discover that they’re pregnant, not seeking treatment because they’ll have to take a drug test. Then what happens from that? Will they be in trouble, or will they be judged, or what happens?*

Similarly, another participant described people in the community “[delaying] care because of fear for judgment during a pregnancy while being a substance…user” and allowing that fear to keep people from going “to the doctor to get prenatal care.”

#### 3.2.3. Cultural/Social Influence on Family Planning

The cultural and social influence on family planning subtheme captures the influence of Appalachian culture and social norms as barriers to Appalachian community members seeking care. In particular, participants discussed religion and “conservative” or “narrow” norms as things that often keep Appalachian community members from seeking family planning services, particularly related to “what women are allowed to do sexually.” One participant stated:

*I’ve heard people in the region talk about their fear of using methods to avoid getting pregnant [because of] religious [reasons]. Religion can keep you, a lot of people can think it’s sinful almost to use birth control or something like that, there’s some intersection there.*

Participants also discussed social norms around childbearing, which can lead women to feel pressure to have children. One participant stated, “people [may] feel pressured to avoid using contraceptives from their significant other or family members to start a family.” Similarly, participants added that when a partner or family member pressures someone to start a family, that person may avoid seeking care because they do not want their family to know that they are using contraceptives. In addition to issues of confidentiality and trust, this point highlights the importance of, as one participant put it, contraceptive methods that “you [can] hide…from your family or your partner.”

#### 3.2.4. Decision-Making Priorities

The decision-making priorities subtheme captures issues related to what Appalachian community members consider important when making family planning decisions, as these priorities inform their desire and ability to seek health services. As one stakeholder put it:

*I think one other thing to consider, especially, is that I think we in this community are so focused sometimes on effectiveness, we forget even maybe about our own experiences with contraception. And we consider lots of things, how will it make my skin look? Or, how it will control menstrual cycles, or how it feels, things like that.*

Participants discussed the many benefits and side effects of various contraceptives, pointing out that people have different needs and priorities. Community members’ individual priorities are influenced by the culture they live in and greatly impact their care-seeking. As such, participants suggested that it would be helpful to have Appalachian community members rank their “top priorities for reproductive healthcare and family planning” or report what they “consider when choosing…contraception.” One participant noted, “You might really be surprised at what comes up to the top. I’m sure effectiveness will, but I’m betting it won’t be the only one.”

#### 3.2.5. Comfort with Reproductive Health Conversations

The comfort with reproductive health conversations subtheme describes the ways that Appalachian community members’ ability to seek or pursue family planning services is related to, and often limited by, their levels of comfort discussing these topics, thereby limiting family planning access by limiting the acceptability of these health services. This issue is linked to culture and norms, which impact community members’ values, their perceptions of their needs and the services being offered, and their comfort with seeking health services. Participants discussed how, because of community norms and values, some Appalachian community members may not be comfortable approaching a healthcare provider to discuss family planning or reproductive health.

In discussing this topic, the stakeholders offered several suggestions about how to help community members reach health services despite norms that may make them uncomfortable seeking care. One participant mentioned a successful program in which mothers and daughters attend together and receive reproductive health information. This participant stated:

*…[the parents] really enjoy having someone else to do the conversation for them. Cause then if the daughters do too, they feel it’s more comfortable. And it opens [the] door where now they are able to ask those questions and see to it.*

Additionally, participants discussed how telehealth and virtual health services can help uncomfortable community members receive care. One participant mentioned that people may be more comfortable using “the internet to explore resources,” and another participant discussed how these resources may be especially helpful for young community members, stating:

*They might be more comfortable with that, especially with your mid-20s, mid-30s audience. If they could just go online, college-age kids can talk to that instead of going to their local health department. They might be willing to ask more personal questions or seek birth control after that. Or seek testing after that.*

Participants discussed Appalachian community member’s overall lack of comfort with reproductive health and family planning topics, suggesting ways that community programs and online resources might be used to increase community members’ comfort and help them access health services. Overall, stakeholders acknowledged that the acceptability of health services is limited in Appalachia when community members are unable to seek or pursue family planning services because their culture, norms, or values create discomfort with reproductive health conversations.

### 3.3. Availability and Accommodation 

The availability and accommodation theme referred to community members’ ability to reach healthcare services. Quotes coded as availability and accommodation denoted the physical existence of health resources, the geographic location of health services, hours of opening and appointment availability, or the availability of transportation as issues that prevented Appalachian community members from being able to physically reach family planning and substance use services in a timely manner.

#### 3.3.1. Provider Availability

The provider availability subtheme captured participants’ descriptions of community members’ inability to reach services because of a lack of reproductive healthcare providers, many of whom work limited hours in a given community. One participant expressed this point, stating:

*One of the things that we have seen here in our rural communities, is access to our providers. While we might have an OBGYN in every county, they’re only in that one county on Monday or let’s say Wednesday, from 10 to 2, and they don’t start taking appointments until Monday, two days beforehand. A lot of times people can’t get in to receive access.*

Participants discussed how this issue is related to state-level “budget cuts for family planning,” which vary based on the state and subregion and can lead to “dropping time for [a] provider,” such that a county may have a single provider who “just comes in once a month.” To this point, participants described patients being “stuck sitting in a waiting room waiting for hours to be seen” because of a lack of access to appropriate reproductive healthcare providers. Participants discussed how these issues are especially problematic in rural areas, where “most of the…abortion clinics have been shut down…even when there were more clinics available, they’re hardly ever in a rural area.” Again, the stakeholders suggested that the use of remote or online health services could help community members in some circumstances bypass the provider shortage and access healthcare.

#### 3.3.2. Available Facilities/Services

Similar to the issue of provider availability, participants reported that a lack of available facilities and services prevents many Appalachian community members from reaching reproductive health and substance abuse treatment services. One participant discussed this problem, stating:

*One of the biggest problems we face with that, is we don’t have a lot of [substance abuse] recovery facilities outside of [the larger counties]. So, when you get into the rural communities, we have support groups, but we don’t have medical staff. We don’t have the recovery facility that we can connect with, where we could implement the [family planning] program, so we’re looking at how we can further the program in the rural communities.*

Similarly, the stakeholders described a lack of available family planning facilities and services, particularly in conservative, rural communities. One participant stated that the nearest abortion clinic is “four and a half hours away from us, and you know you can’t put a brick and mortar clinic here. God knows you can’t do that close to a Southern Baptist around here.” Another participant discussed the influence of policies which impact the availability of family planning services and make reaching healthcare services more difficult, stating that it is:

*…important to think about how people might have to cross state lines, and then what state laws also affect their access to family planning, or how their insurance factors into where they have to go, based on if their closest clinic might be across state lines, or if they have to travel further to still use state Medicaid or something like that.*

#### 3.3.3. Reaching Health Services

In addition to issues with provider and service availability, the stakeholders reported that Appalachian community members may have trouble reaching reproductive health and substance use services. This subtheme refers to barriers to reaching these services that are related to how far away appropriate services are and a lack of reliable and accessible transportation to reach those services. Participants discussed the difficulty of reaching reproductive healthcare when affordable services are located far away, with one participant stating:

*…because when you’re looking at Appalachia, you are looking very much at rural areas where it’s not right across the road, necessarily, to get to a healthcare provider. When you are looking at affordable options, how far are you, or what distance are you to a healthcare provider, whether it be a health department or free, or affordable services.*

Another participant described how difficult it is for pregnant women in their community, especially those who use substances, to reach care, stating:

*…the local hospital stopped delivering babies. Women could be seen for prenatal care, and then they had to travel at least 45 min to over an hour, depending on where they lived, to actually deliver in a hospital…for high risk, they had to travel [even farther]. Some women, particularly those women with addiction issues, don’t have transportation. They didn’t have cars. They didn’t have resources.*

Similarly, the stakeholders discussed various transportation options and the barriers to each for Appalachian community members. They argued for the importance of understanding community members’ access to transportation, with one participant stating, “I think finding out about people’s transportation, and the access that they have is important. Because, especially in our area, we don’t have [an] alternative.” Participants mentioned taxi and ambulance services as possible “transportation options for people without cars, or without money for gas,” but acknowledged the cost of these options and the difficulty of accessing them, with one participant acknowledging that arranging transportation can be challenging by stating that there are “always catches to how you can request [transportation services], and what [exactly] you can request.”

### 3.4. Affordability

The affordability theme referred to community members’ ability to pay for reproductive health and substance use services. Quotes coded as affordability denoted direct and indirect costs, including opportunity costs, or the lack of appropriate income, social capital, lack of health insurance coverage, or responsibility for co-insurance payments as factors that prevented community members from being able to pay for reproductive health and substance abuse treatment services. These issues related to Appalachian community members’ economic capacity to use time and/or resources on healthcare.

#### 3.4.1. Direct Costs of Services

The direct costs of services subtheme captured participants’ descriptions of Appalachian community members having difficulty affording reproductive health and substance abuse recovery services because of a lack of affordable options, low incomes, or a lack of health insurance. One participant noted affordability as a problem, stating, “financial questions, I think are the thing that consistently comes up for that region.”

Participants specifically mentioned the importance of asking community members, “Can you afford contraception?” because they frequently encounter this barrier to care among people in their area. Another participant mentioned that unplanned pregnancies in the Appalachian region often occur because community members have “no insurance.” Additionally, participants discussed cost as a barrier to abortion services, describing policy barriers that prevent federal or state funds from covering these health services. The stakeholders also described the power of abortion funds (i.e., charitable organizations that provide financial aid to help people pay for unaffordable abortions [41]) to help community members afford abortion care, with one participant describing the importance of assessing whether Appalachian community members “know if they do qualify for [abortion] funds,” following up that some agencies in the area address affordability of abortion services by “screen[ing] everybody for funding for their abortions.”

#### 3.4.2. Indirect Costs

In addition to direct costs of health services, there are indirect costs associated with reproductive healthcare and substance use treatment, including paying for transportation to health facilities and pharmacies. Participants stated that “the cost of transportation” can prevent Appalachian community members from seeking healthcare. They also noted that healthcare options are often scarce for community members with limited transportation resources while people with financial resources have the luxury of choosing from multiple healthcare options. Along this line, one participant stated, “You have that option if you have the means to go there [to access services that are far away] and get that.” Additionally, participants described working with community members whose lack of resources led them to risk their reproductive health in order to afford transportation to health services and to meet other basic needs, as sex was one of the only resources that they had access to. As one participant stated:

*A lot of the women that I worked with who were experiencing homelessness and had issues with addiction, also used sex as a currency. To get drugs, to get a place to stay, to get a ride to the clinic, whatever the case may be.*

### 3.5. Appropriateness

The appropriateness theme referred to community members’ ability to engage in healthcare services. Quotes coded as appropriateness mentioned the technical and interpersonal quality of healthcare, the adequacy of care, care coordination and continuity, lacking feelings of empowerment, or the type of information provided to community members as issues that prevented people from being able to engage with reproductive health and substance use services. These issues related to the fit between the services being provided and Appalachian community members’ needs as well as the level to which patients are involved in decision making and treatment decisions.

#### 3.5.1. Patient-Service Fit

The patient-service fit subtheme captured Appalachian community members’ ability to engage with healthcare providers and the services they provide. Patient-service fit can become a barrier to people’s ability to engage when providers misunderstand or ignore the needs of the communities they serve. For example, participants discussed how providers in Appalachia often do not understand the needs of gender and sexual minorities, reporting that “for transgender men and gender non-conforming folks, …their identity [may have] prevented them from being able to access contraception.” In this example, there is a mismatch between the family planning needs of gender minority patients and the available services, leading to an inability for these patients to engage in appropriate health services.

Participants also focused on the needs of Appalachian community members who use substances but also need reproductive health and family planning services. They mentioned that healthcare providers may fail to acknowledge or address these needs with their patients, thereby preventing community members from engaging in necessary healthcare. One participant discussed the importance of medical professionals addressing substance use with pregnant patients “when they do engage in those kinds of behaviors when they’re pregnant, whether it be opioid use or smoking, or drinking, or tobacco usage of any kind.” Participants also discussed a similar problem in which healthcare providers may offer either reproductive health or substance use recovery services but are ill-equipped to provide both services. The stakeholders noted the inappropriateness of this service gap, especially in the context of the opioid crisis. As one participant stated:

*We [in our community] have a program…helping lower the incidences of neonatal abstinence syndrome. And what we learned in the research was the recovery facilities had provided very little information to women who were seeking help through the recovery facilities, to talk about preventing pregnancy. In fact, we actually had several homes tell us that, that was outside of their wheelhouse. And we were like, ‘How can that be outside of your wheelhouse?’ If you’re not telling women how to prevent an unintended pregnancy, and they’re coming up pregnant, and there’s all these pieces that go along with it. And I said, ‘What do you do when they do when they do come to you and they’re pregnant?’ They’re like, ‘Oh, we kick them out of our program.’ And I’m like, ‘What?!’ It’s kind of crazy.*

#### 3.5.2. Provider Bias

The provider bias subtheme captured stakeholder descriptions of Appalachian community members’ inability to engage adequately with reproductive healthcare or substance use services when providers do not involve patients in decision making and treatment decisions and instead limit patient autonomy by making assumptions about or even coercing patients into participating or not participating in certain health services. Participants discussed this issue as often related to social norms, providers’ personal values and expectations, and misinformation among providers. One participant discussed their experience with healthcare providers who believe that long-acting reversible contraception (LARC), such as an intrauterine devices (IUD), is the most appropriate family planning service for certain groups of people, regardless of patient desire for this contraceptive method. As this participant stated:

*I think it’s much more related to what their [providers’] values are. Sometimes you will have providers that are really committed to wanting to provide good access to all people, regardless of substance use or not. And then you have other providers who maybe will identify and say things like, ‘You know what? [They are a] drug addict, [so] I’m going to push IUDs.’ I’ve heard providers say that. Or, ‘[For] people that are in poverty, I’m going to push IUDs, because I don’t want them to have more kids.’ I don’t think it’s necessarily what their job description is, as much as what their values are. If they’re having a high…[degree of] values against those people, that might affect access.*

This participant described certain populations (e.g., people who abuse substances, people living in poverty) that healthcare providers may coerce into using LARCs because they consider these to be populations for which reproduction is undesirable.

Many participants also described healthcare providers “refusing to provide” abortion services as well as other family planning services, such as birth control or emergency contraception, that “they would potentially consider an abortive agent.” As one participant stated, “it might be surprising to find out how many people [are] just actually being flat out denied something they went and asked for.” In addition to refusing to provide requested services, participants discussed healthcare providers in Appalachia who refuse to even mention certain services that they disagree with as potential options for their patients. As one participant stated, “They [healthcare providers] won’t even discuss that [abortion] as an option. They won’t even say you have that option.” Similarly, another participant stated, “there are some physicians that still believe contraception is abortion, so they don’t want to offer it to patients, and they won’t offer it to unmarried patients.”

Participants also described how reproductive healthcare providers sometimes allow their assumptions about their patients to drive which services they offer. In one example, a participant stated:

*I have [an overweight] friend once who went to a gynecologist. And when she asked for birth control, the gynecologist looked at her and said, ‘why?’ And she’s like, ‘I’m not sure what you’re...what do you mean why? Because I’m sexually active.’ And the gynecologist actually responded and said, ‘You’re sexually active?’ Like [the gynecologist was] so shocked that a fat person could have sex. But that’s what we’re dealing with.*

Here, in a particularly salient quote due to the high rates of obesity in the Appalachian region [18], a provider allowed their own bias about the sexuality and reproductive health needs of an overweight patient to influence the health services offered to this patient, potentially compromising the patient’s ability to engage in appropriate health services. Thus, the provider bias subtheme refers to Appalachian community members’ inability to engage with health services when healthcare providers’ personal values or biases cause them to provide inappropriate or inadequate services.

#### 3.5.3. Provider Training

The stakeholders also related Appalachian community members’ ability to engage in reproductive health and substance use services to the training and education that their healthcare providers had obtained, discussing how a lack of adequate “provider preparation” can prevent providers from being able to provide appropriate health services. Participants specifically related this issue to provider training about how to provide family planning services. One participant stated, “it seems like family planning is being handed to the primary care providers now, rather than the specialists or the family planning centers.” Participants discussed this as an issue particularly because primary care providers may not be adequately prepared to offer family planning services. As one participant stated, “There is little to no training that’s happening in general around contraceptive counseling…even in medical school.” Participants discussed their concern that ill-prepared healthcare providers were being expected to be primary family planning healthcare providers, which can lead to Appalachian patients receiving poor reproductive healthcare.

Additionally, participants described populations whose needs may not be covered in healthcare provider education, leading to community members who are unable to engage in appropriate healthcare because their providers are not adequately prepared to meet their needs. One example is healthcare providers who are not prepared to work with patients who are transgender. One participant discussed this issue, stating:

*So, I’m pretty sure that she probably assumed at that point that [my transgender friend] was gay…I don’t think she knew how to react with her at that point. She’s transgender, she’s not gay. But still, I think it just makes things very complicated that she...I think it is an issue of preparation. Like are our providers really prepared?*

## 4. Discussion

These findings describe community stakeholders’ perceptions of the family planning practices in Appalachia in the context of the opioid epidemic. Barriers to obtaining care were categorized using overarching a priori themes from Levesque et al.’s (2013) healthcare access framework [4], and multiple subthemes were identified to further define, and therefore better understand, these needs. This is a unique contribution to the knowledge base, as this framework has not been used previously to categorize community member perspectives about family planning services or the impact of community-level substance use on such services. Taken together, these results illustrate that community stakeholders perceive an interconnected fabric of barriers to seeking family planning and substance use treatment services in Appalachia that has never been previously explored and highlight stakeholders’ perceptions of a lack of existing knowledge about if and how these obstacles are overcome.

### 4.1. Approachability

The identified themes on approachability highlight the need for greater reproductive healthcare transparency, which refers to the “open, honest, and accessible” [42] (p. 33) communication of information about the price and quality of services to patients and community members [25,37]. As has been recommended by others for general healthcare, providing approachable services involves disseminating health information online, as community members are increasingly using the internet to seek health information; this is particularly applicable in Appalachia, where the internet and social media are often used as sources of health-related information [16,25,43]. Our results also speak to the need for health information that is appropriate to the culture, reading level, and health literacy of its intended targets, which is consistent with recommendations from primary care providers in relation to other rural areas [44]. These findings are also consistent with earlier research specific to Appalachia, showing that lower health literacy among Appalachian adults and the existence of inappropriate health messaging are barriers to accessing care in the region [24,45,46]. Research also indicates that a lack of knowledge about healthcare and cultural beliefs can be a major barrier to healthcare, as evidenced by results from a study indicating that Appalachian residents shared misconceptions and beliefs about the human papillomavirus (HPV) infection, which prevented them from accessing appropriate related healthcare [43].

### 4.2. Acceptability

In relation to acceptability, our results suggest that Appalachians may lack trust in healthcare providers and confidentiality in relation to family planning services. This is consistent with previous research on other types of healthcare indicating that Appalachians report a lack of trust in healthcare providers, which impacts their desire to seek healthcare [16,22]. Stakeholders in our study also described community social norms and religious expectations about gender roles, childbearing, and contraceptive use that may create community pressure to adhere to certain behavioral expectations (e.g., not use birth control, continue an unplanned pregnancy). This finding is similar to the results of other research indicating that Appalachian community members avoid healthcare services due to fear of stigmatization, and because of social, cultural, and religious beliefs that place a high value on self-reliance and closed/close-knit communities [15,20,47,48]. The findings are not surprising in light of research indicating that culture impacts health beliefs and health service use in other settings with other populations [49]. Understanding these cultural priorities specific to Appalachia is key to helping Appalachian community members feel comfortable seeking family planning services and helping providers deliver appropriate reproductive healthcare.

### 4.3. Availability and Accommodation

In relation to availability and accommodation, our findings indicate that focus group participants perceive that Appalachians in their communities often lack the ability to reach reproductive health and substance use treatment services due to a lack of provider availability and a scarcity of geographically-close health facilities and services, which requires people to travel far distances to seek care. This difficulty is exacerbated by the lack of public transportation infrastructure in most areas of the Appalachian region. This finding is consistent with other results indicating that when fewer substance abuse treatment facilities are accessible to Appalachian women, they will be less likely to use treatment services at all [50], and a study conducted by LeGrow et al. (2007), which showed that Appalachian residents who reported transportation problems also described difficulty accessing healthcare [51]. Similarly, others have found that residents of rural communities utilize healthcare services more when they have access to a form of transportation, such as possessing a driver’s license or having transportation through a friend or family member [52]. Research also suggests that those living long distances from abortion providers are more likely to be at an advanced gestational stage at the time of abortion obtainment when compared to abortion patients who live closer to a provider [53]. People in rural communities have been shown to experience similar problems in accessing prenatal and intrapartum care, as Grzybowski and others (2011) showed in their study where patients who had to travel farther distances for care experienced higher rates of health complications when in labor [54].

While expanding transportation services can be challenging for rural communities, research indicates that one of the primary reasons that community members report needing public transportation is to access health services [55]. Alternatively, Appalachian communities have benefited from providing reproductive healthcare via telehealth communication. For example, administering reproductive health services via telemedicine to a sample of adolescent young women resulted in increases in condom use and HPV vaccination [56]. Thus, while developing infrastructure for transportation services could overwhelm rural communities, alternative methods such as telecommunication may also decrease gaps in services for Appalachian community members.

### 4.4. Affordability

In relation to affordability, our results indicate a lack of health insurance among Appalachian residents as a barrier to healthcare affordability as well as a lack of economic capacity for covering the indirect costs of accessing healthcare, such as transportation or childcare. This finding is in line with existing research showing a lack of health insurance in Appalachia as a barrier to accessing general healthcare [26]. The problems with affordability are likely related to a lack of insurance coverage, or high out of pocket co-insurance payments. The highest rates of uninsurance occur in states that opted out of expanding Medicaid coverage through the Affordable Care Act, or in states that have connected work requirements to the availability of Medicaid coverage [32,57]. Given the high rates of poverty in areas of Appalachia and the rurality of many communities, a lack of transportation combined with long travel distances to providers has led to an affordability crisis for health services overall, with reproductive healthcare and family planning services, in particular, being very difficult to access [58]. Research suggests that potential methods of increasing healthcare affordability include creating partnerships with existing community resources, such as nonprofits and grassroots organizations [59].

### 4.5. Appropriateness

These results around appropriateness describe an inadequate fit between the needs of the individual and the services provided, including both perceived and actual mismatches in interpersonal aspects of the patient-provider relationship, such as provider bias and provider training. This is illustrated by gaps in the available services and service needs of the community, such as a lack of substance use recovery services for pregnant people and a lack of family planning services for those receiving substance use treatment. Given the elevated rates of opioid use among individuals of reproductive age in this region, as well as the increases in NAS, this inappropriate gap in service coverage offers a stark example of a missed opportunity for community-centered service provision. We recommend co-located family planning and substance use treatment services, similar to co-located prenatal care and substance use treatment services that have shown positive outcomes when delivered in an integrated format [34,35].

When services are available, our results also describe multiple forms of provider bias experienced by those seeking family planning services. Some participants reported perceptions that providers may be coercing patients into specific forms of contraception (i.e., LARCs) if they were low-income or using substances, while others discussed perceiving providers refusing to counsel on abortion or offer contraception or emergency contraception, due to providers’ personal beliefs. These accounts fit into a larger narrative regarding provider bias related to culture and/or social class in the family planning setting, which is particularly problematic given a history of provider-perpetuated reproductive injustices, such as involuntary and coerced sterilization, often perpetrated against marginalized populations [36,38]. Evidence suggests some community members, particularly the economically disadvantaged and people of color, remain wary of public health initiatives and family planning counseling that pushes LARCs and voluntary sterilization [36,38]. This issue is amplified by a lack of cultural compatibility between providers and the communities they serve, as recruiting and retaining diverse healthcare providers and support staff can be challenging [60]. Refusing to counsel or offer certain types of care, such as contraceptive prescriptions or abortion referrals, illustrates a form of provider bias that has recently been legitimized by the expansion of federal protections under the “conscience clause” [61]. Along the lines of a more patient-centered care strategy, the literature suggests that patients desire autonomy in reproductive decision making, indicating that healthcare providers should be offering comprehensive options and assisting their patients in decision making rather than limiting options based on individual provider bias [62].

Our results also touched upon the lack of adequate provider training in culturally-competent care for gender and sexual minorities living in Appalachia. As discrimination is a known barrier to accessing care for gender and sexual minorities, and it is thought to drive poorer health utilization and outcomes in these populations, adequate provider training is essential to combat these shortcomings [63]. Also described was a perception that primary care providers lacked the necessary training to provide family planning services, which creates a barrier for people to access family planning services as it undermines trust in the quality of the care received. This finding is supported by existing literature indicating that healthcare providers lack adequate reproductive health and family planning knowledge and often fail to provide family planning information to patients of reproductive age [44,64,65]. In order to address these issues, primary care providers should be adequately trained in family planning and prepared to offer these services, especially in rural areas where there may be limited access to dedicated family planning providers.

#### Strengths and Limitations

The purpose of qualitative research is to deepen understanding rather than establish statistical-probabilistic generalizability [66]. As such, we considered the types of qualitative generalizabilities proposed by Smith (2018), concluding that we have provided evidence of analytical generalization [66]. Analytical generalization refers to generalizing a set of results to an established theory or concept or using different methodology to reexamine established concepts and theories [66]. This study has used qualitative methodology to apply Levesque et al.’s (2013) existing healthcare access framework to family planning in Appalachia in the context of the opioid epidemic, thereby establishing analytical generalization through the application of this framework to our framing of these findings.

One main limitation of the study is the relative homogeneity of the focus group participants. Participants were professionals who live and have a stake in the health of Appalachian communities, yet who are also gainfully employed in the community, and thus they did not represent community members from populations that would be considered more vulnerable, such as homeless residents, or those who are actively using opioids or other substances. Therefore, future research should be designed to gain perspectives about these topics from a wider variety of vulnerable populations, and we are currently engaged in ongoing research to address some of these limitations.

## 5. Conclusions

The results of this study characterize the perspectives of Appalachian community stakeholders in regards to what those stakeholders perceive about family planning practices in their communities and how those practices may be impacted by the increased rates of opioid use that these communities have been experiencing in recent years. These results unsurprisingly reflect stakeholders’ concerns that family planning and substance use treatment services are often provided in silos, potentially creating a service gap that does not adequately provide for the needs of the whole person. Experts suggest that people are interested in receiving family planning and substance abuse treatment in the same program [67], yet, stakeholders in our study report perceiving few existing opportunities for this type of service integration in their own communities. Stakeholders identify many factors that they believe coalesce to create conditions that impede Appalachian community members from seeking and obtaining appropriate reproductive health and addiction care.

Some elements of the major and sub-themes of the results are not unique to the experiences of Appalachian residents, however. For example, lack of health literacy is a known barrier to accessing healthcare in a variety of vulnerable populations and settings, and scarcity of healthcare providers has created systemic obstacles to accessing care in many different and geographically-diverse communities [68,69]. Other aspects of these findings may be considered unique to the region or specific to family planning or substance use services, which is the main contribution of this study to the knowledge base. For example, our results describe cultural values found in communities of Appalachia that may have a negative influence on seeking family planning care, such as the high value placed on childbearing or religious views that stigmatize contraception use. Our results also suggest some barriers unique to the socio-political environment of Appalachia, namely the presence of state-level health policy in some Appalachian states that limits access to reproductive health services, particularly in rural areas of Appalachia, where these services are already sparse.

These findings highlight the stakeholders’ perceptions that there is not enough existing knowledge about how women in Appalachia navigate family planning practices in all five of the above aspects of healthcare access. The overlapping and cumulative burden of barriers to family planning and substance use care in Appalachia are formidable, and these results suggest that tailored messaging and programming across disciplines and networks in the region is urgently needed. We plan to use the findings from the current study to inform additional research to further study family planning needs, in the context of the opioid epidemic, throughout the Appalachian region.

## Figures and Tables

**Table 1:** Description of a Priori Themes and the Sub-Themes That Emerged.

Themes ^1^	Description of Themes ^1^	Sub-Themes
Approachability	The ability to perceive that services exist, can be reached, and are needed. Related to the transparency of services, providers’ screening and outreach activities, information about available services, and community members’ health literacy, health beliefs, expectations, and trust in providers.	Treatment services for people abusing substances
		Appropriate outreach materials
		Appropriate health knowledge: Knowledge about family planning Knowledge about accessing services Source of health information
Acceptability	The ability to seek healthcare. Related to cultural and social factors that determine whether people accept health services.	Trust in healthcare providers
		Stigma/fear of judgment
		Cultural/social influence on family planning
		Decision making priorities
		Comfort with reproductive health conversations
Availability and Accommodation	The ability to physically reach health services in a timely manner. Related to the geographic location of community members and services, the hours that health facilities are open, the availability of appointments, the accessibility of the facilities, and people’s access to transportation and social support.	Provider availability
		Available facilities/services
		Reaching health services
Affordability	The ability to pay for health services. Related to the price of care and community members’ income, assets, social capital, and economic capacity to spend time and resources on health services.	Direct costs of services
		Indirect costs
Appropriateness	The ability to engage with healthcare services and healthcare providers. Relates to the fit between services and clients’ needs, the interpersonal quality of services, the adequacy of provider training, issues of provider bias, and the patient’s ability to participate in decision making and treatment decisions.	Patient-service fit
		Provider bias
		Provider training

^1^ Adapted from Levesque, Harris, and Grant Russell, 2013 [4].
